# Data for the industrial and municipal environmental wastes hazard contaminants assessment with integration of RES2D techniques and Oasis Montaj software

**DOI:** 10.1016/j.dib.2020.106595

**Published:** 2020-11-28

**Authors:** Mohd Hariri Arifin, John Stephen Kayode, Khairel Izzuan Ismail, Manan Abdullah, Asha Embrandiri, Shahidah Mohd Nazer, Azrin Azmi

**Affiliations:** a Program Geologi, Pusat Sains Bumi dan Alam Sekitar, Fakulti Sains dan Teknologi, Universiti Kebangsaan Malaysia, 43600 Bangi, Selangor, Malaysia; b Department of Research and Innovations, Shale Gas Research Group, Universiti Teknologi PETRONAS, Institute of Hydrocarbon Recovery, Persiaran UTP, 32610 Seri Iskandar, Perak Darul Ridzuan, Malaysia; c Geo Technology Resources SDN BHD. 31-1, Jalan Mawar 5B, Taman Mawar, 43900 Sepang, Selangor, Malaysia; d Department of Environmental Health, College of Medicine and Health Sciences, Wollo University, Dessie P.O. Box 1145, Amhara, Ethiopia

**Keywords:** Industrial and municipal wastes assessment, Environmental hazard, Waste contaminants volume, Pollutants, Kepong, Kuala Lumpur, Peninsula Malaysia

## Abstract

Industrial, and municipal wastes are part of the main sources of environmental hazards as well as groundwater and surface water pollutions. If not well composed, treated, and safely disposed, it could permeate through the subsurface lithologies by reaching down to the underground water aquifers, particularly in zones of unprotected aquifer units. Pollutants, most especially the landfills leachates that encompassed organic contaminants, ammonia, nitrates, total nitrogen, suspended solids, heavy metals and soluble inorganic salts, i.e., soluble nitrogen, sulphur compound, sulphate and chlorides, could posed undesirable environmental impacts due to inappropriate disposals that may give rise to gaseous fumes and leachate formations. An electrical resistivity geophysical technique utilizing the RES2D no-invasive, cost-effective and rapid method of data collection was integrated with the 3D Oasis Montaj software to approximate the volume of the generated rectangular prism model of the contaminants delineated from mixtures of the industrial, and municipal wastes plumes to be 312,000 m ^3^.

## Specifications Table

**Table:** 

Subject	Waste Management and Disposal
Specific subject area	Electrical Resistivity Tomography
Type of data	Excel Table Image Figure
How data were acquired	Data collection was carried out with a Two-Dimensional Electrical Resistivity Tomography (ERT) data acquisition technique utilizing 41 stainless steel metal electrodes, connected to a 100 m length multicore cables through the copper connecting jumpers. ABEM Terrameter SAS4000 resistivity meter system with inbuilt microprocessor, together with an electronic switching ABEM LUND ES 10 - 64C selector controlled circuitry unit, were used to auto select the relevant four electrodes for each station point measurement. Wenner-Schlumberger electrodes configuration were conducted with 200 m length at 5 m inter-electrode spacing.
Data format	Raw Analyzed Filtered
Parameters for data collection	The RES2DINV inverse modelling software for the Two-dimensional model sections of the subsurface layer resistivity were prepared, to generate RES2-D pseudo-sections data that defines a bi-dimensional model of the subsurface lithologic layers, using distance against the estimated vertical depths variations obtained from the geoelectrical inverted resistivity data. The inversion RES2D ERT data files, the coordinates, and elevations of each electrode points along the survey lines were compiled together to produce the 3-D model using the Oasis Montaj Software© that helped to generate the approximate contaminants volume of the prism shape.
Description of data collection	The six Electrical Resisitivity Tomography (ERT), survey profiles 1-3 were acquired in the East-West directions, and profiles 4-6 vertically arranged along the North-South directions respectively. Four active stainless metal steel electrodes auto selected by the selector throughout the measurement. The data were acquired through parallel survey lines at intervals closely selected for a nominal inter-electrode spacing to evade missing some of the key targets, (spacing n = 13), for 200 m electrode spread. The multi-core electrical signal cables set up for the data acquisition, has an inbuilt mechanism to prevent momentary eddy currents that produces capacitive-inductive coupling between the subsurface layers, and the metal steel electrodes when the injected current passes through. This was done to overcome significant reduction in the contact resistances between the metal steel electrodes and the subsurface layers. A 12V, 45 Amp-hour dc battery was used to supply the current to the Equipment, while its output current intensity was set at 50-100 mA for the data acquisition. This is to facilitate adequate injected current through the 41 stainless metal steel electrodes to penetrate the wastes materials into the subsurface stratum.
Data source location	Institution: Federal Territory, Kuala Lumpur City/Town/Region: Taman Kepong Indah (TKI), Kuala Lumpur Country: Peninsula Malaysia Latitude: N 3° 13′ 46.79" Longitude: E 101° 38′ 25.86"
Data accessibility	https://data.mendeley.com/datasets/4jvf24rt26/1
Related research article	M.H. Arifin, J.S., Kayode*, M.K.I. Ismail, A. M. Abdullah, A. Embrandiri, N.S. Nazer, A. Azmi, Environmental hazard assessment of industrial and municipal waste materials with the applications of RES2-D method and 3-D Oasis Montaj modeling: A case study at Kepong, Kuala Lumpur, Peninsula Malaysia. J. Haz. Materials. https://doi.org/10.1016/j.jhazmat.2020.124282

## Value of the Data

• RES2D, Electrical Resistivity Tomography was integrated with the 3D Oasis Montaj software to quantify the volume of industrial, and municipal wastes contaminants is a novel technique adopted in the data acquisition.
• The usefulness of the data is in the speedy subsurface evaluation for remediation planning, policy formulations on the industrial and municipal waste contaminants for careful plans regarding the environmental impact on the area, by the Environmental Scientists, Municipal Policy Managers, Engineers, and Developers.
• The six RES2-D inversion profiles, helped to classify the subsurface layers into three zones on the basis of the varied resistivity data at a known depth e.g., the soft layers, which encompasses the waste materials, the consolidated layers, and the bedrock.
• A non-invasive, cost-effective and rapid technique of data collection was carried out to improve the data quality while probing depths to the landfill leachates, and contaminants plume for pollution free environments, and preservation of groundwater aquifers, surface water bodies, and the eco-systems.
• The data provided advantages of rapid leachate and contaminants flow monitoring, and demarcation of the potential environmental hazards (PEH), posed to human lives, the engineering surface structures, eco-systems, and groundwater aquifers underlain the Kepong area which could be applicable in any region globally.

## 1 Data Description

**Fig. 1:**
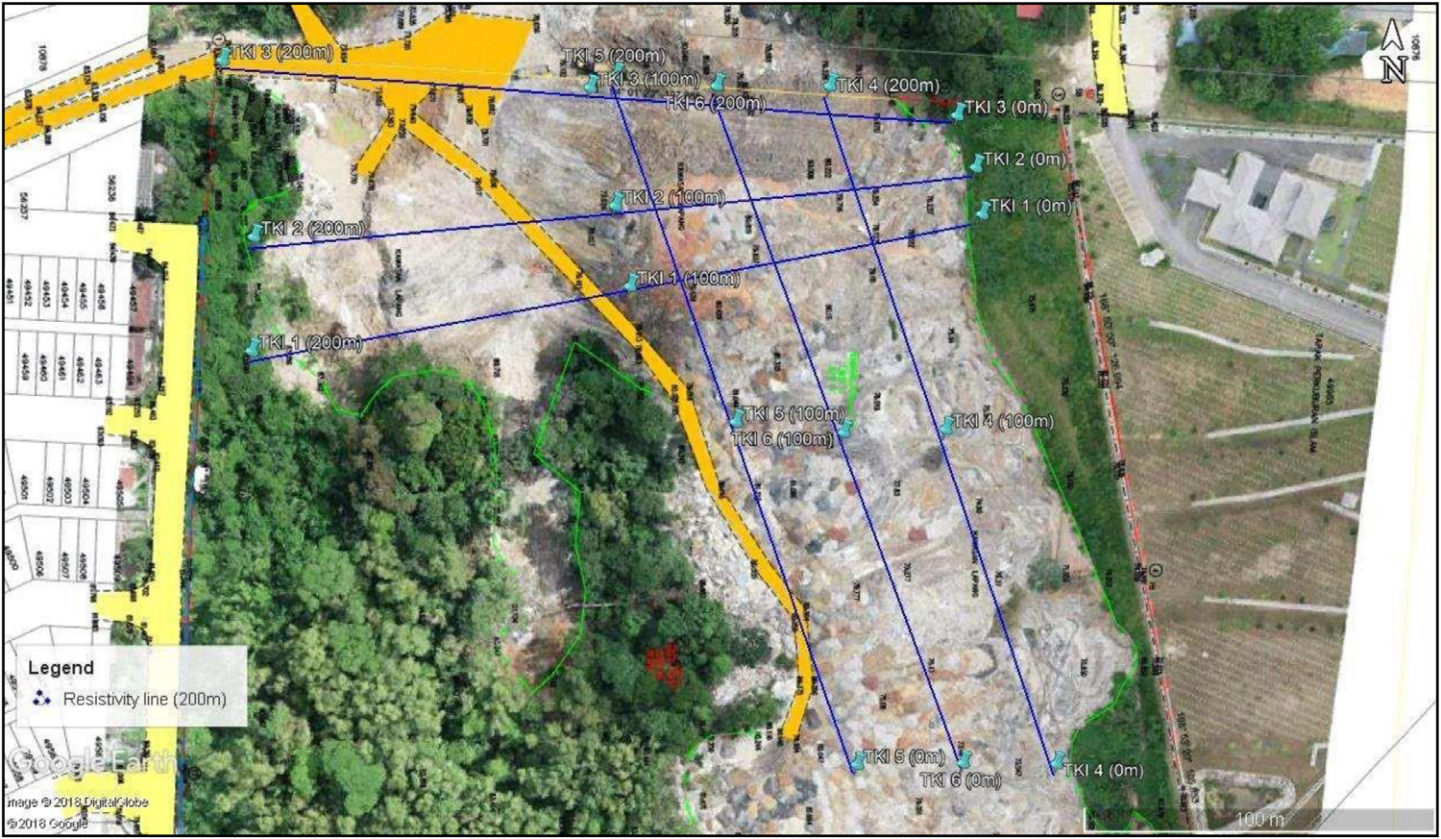
Location map showing the geophysical survey lines, at the Taman Kepong Indah (TKI), Kuala Lumpur, Malaysia, overlaid on the Google Map. Modified after [1].

The area covered by the data is located at about 1.5 km from the Malaysian Institute of Forest Research and about 1 km from the Bukit Lagong foothill. The site elevation profile ranged from between about 80 m to about 101 m above mean sea level. The data location is shown in Fig. 1, comprises of six RES2D ERT survey lines plotted on Google Earth image [1]. The parameters adopted for the data acquisition of the six geophysical survey lines are presented in Table 1. The detail RES2D ERT raw dataset are presented as supplementary file in excel format. The Global Positioning System (GPS) was used to record the coordinates, and elevation of each electrode position, as shown in the supplementary file in a tabular format.

**Table 1:** Data acquisition survey parameters along the six RES2D profiles at the Taman Kepong Indah, Kuala Lumpur.

Survey Line	Electrode Array	Length of Profile (m)	Electrode Spacing (m)	Depth of penetration (m)
TKl1	Wenner-Schlumberger	200	5	35
TKl2	Wenner-Schlumberger	200	5	35
TKl3	Wenner-Schlumberger	200	5	35
TKl4	Wenner-Schlumberger	200	5	35
TKl5	Wenner-Schlumberger	200	5	35
TKl6	Wenner-Schlumberger	200	5	35

**Fig. 2:**
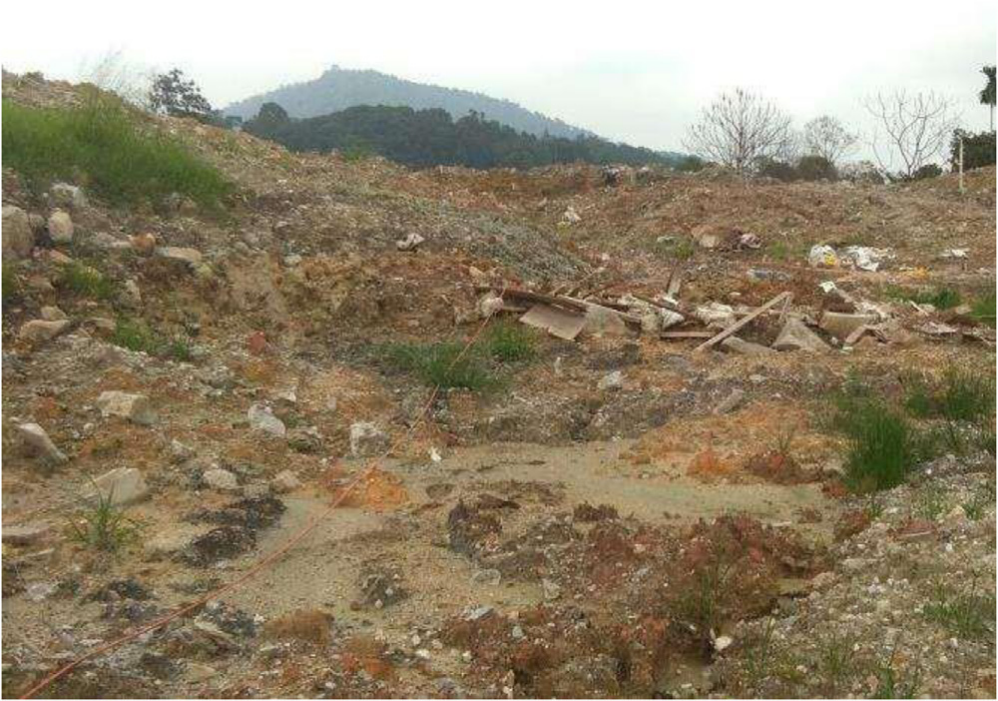
Study site showing the IWM mixed with the MSW.

Activities of industrial waste desposal (IWD) combined with municipal waste materials (MWM), severely contaminated the area, and hence, constituted as source of environmental hadzard, surface water and grounwater polutions, through the contaminants plumes seeping into the underground water bearing structures. The waste materials, are principally made of mixtures from moist cemented soil, and soft industrial wastes materials (IWM) mixed with junks from MWM [1]. Environmental impact of the disposal activities are vissibly notice at the site, (i.e., Fig. 2), as the eco-systems in the area have been subjected to massive destruction from the landfill leachates contaminants plumes.

Established engineering structures, the environments, and eco-systems at the earth's surface, are highly subjective to the subsurface geological conditions [2], [3], [4]. On account of these interconnectivities, successful land-use, environmental planning, and monitoring, relies on the accurate delineation of the near-surface structures, most importantly, in areas characterized by the mixtures of industrial waste materials (IWM), and municipal solid wastes (MSW) [1].

**Fig. 3:**
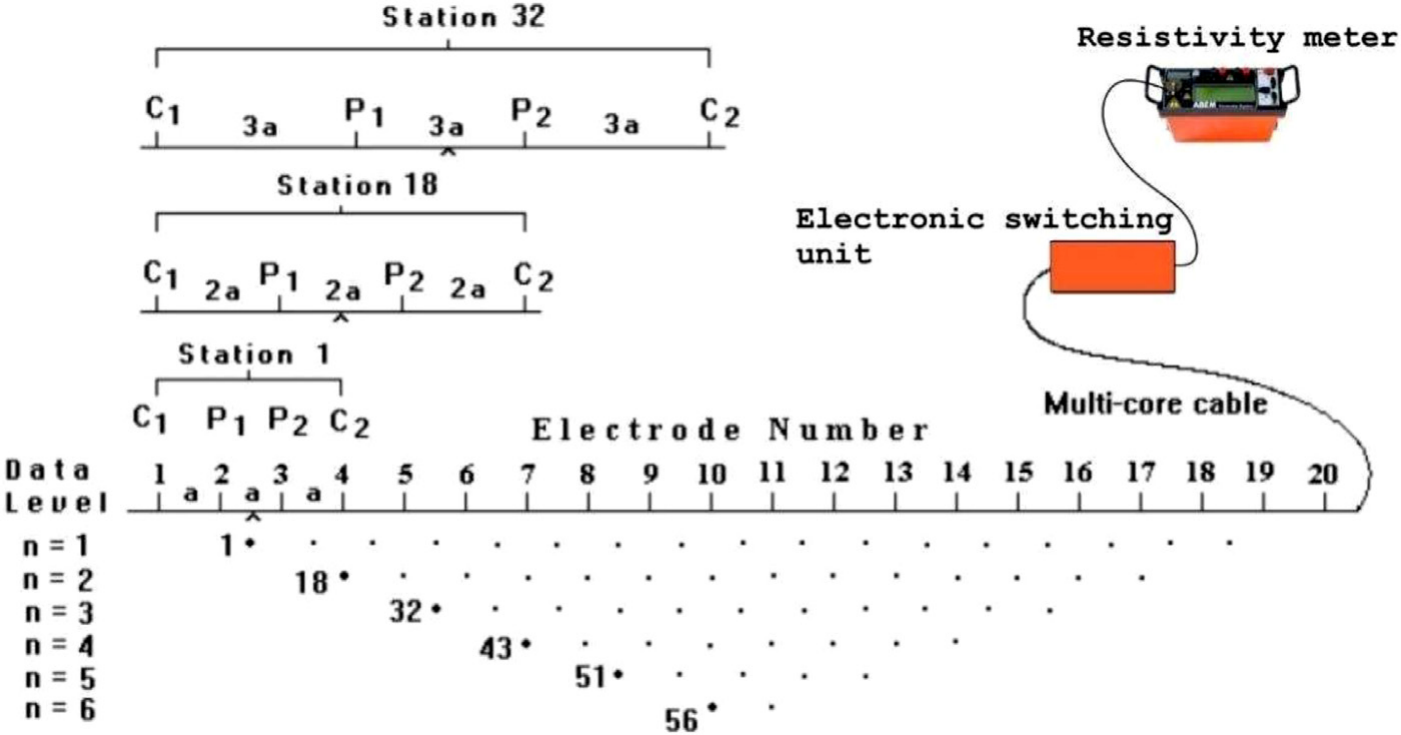
Typical electrode configuration, and sequence of measurement to build up pseudosection Wenner-Schlumberger array modified after [4].

ABEM Terrameter SAS4000 together with ABEM LUND ES 10 - 64C, the electronic switching selector unit, (i.e., Fig. 3), was deployed to carryout the data acquisition using Wenner-Schlumberger electrodes configuration array. The electrodes configuration as shown in Fig. 3, present the sequence of measurement along the six (6) RES2D ERT survey lines used to develop the pseudosection generated from the Wenner-Schlumberger array [4]. The electrodes configuration determined the apparent resistivity measured which is a function of the quantity of electric current injected through the metal electrodes to the subsurface stratum and the resultant potential difference dropped across these electrodes [4].

**Fig. 4:**
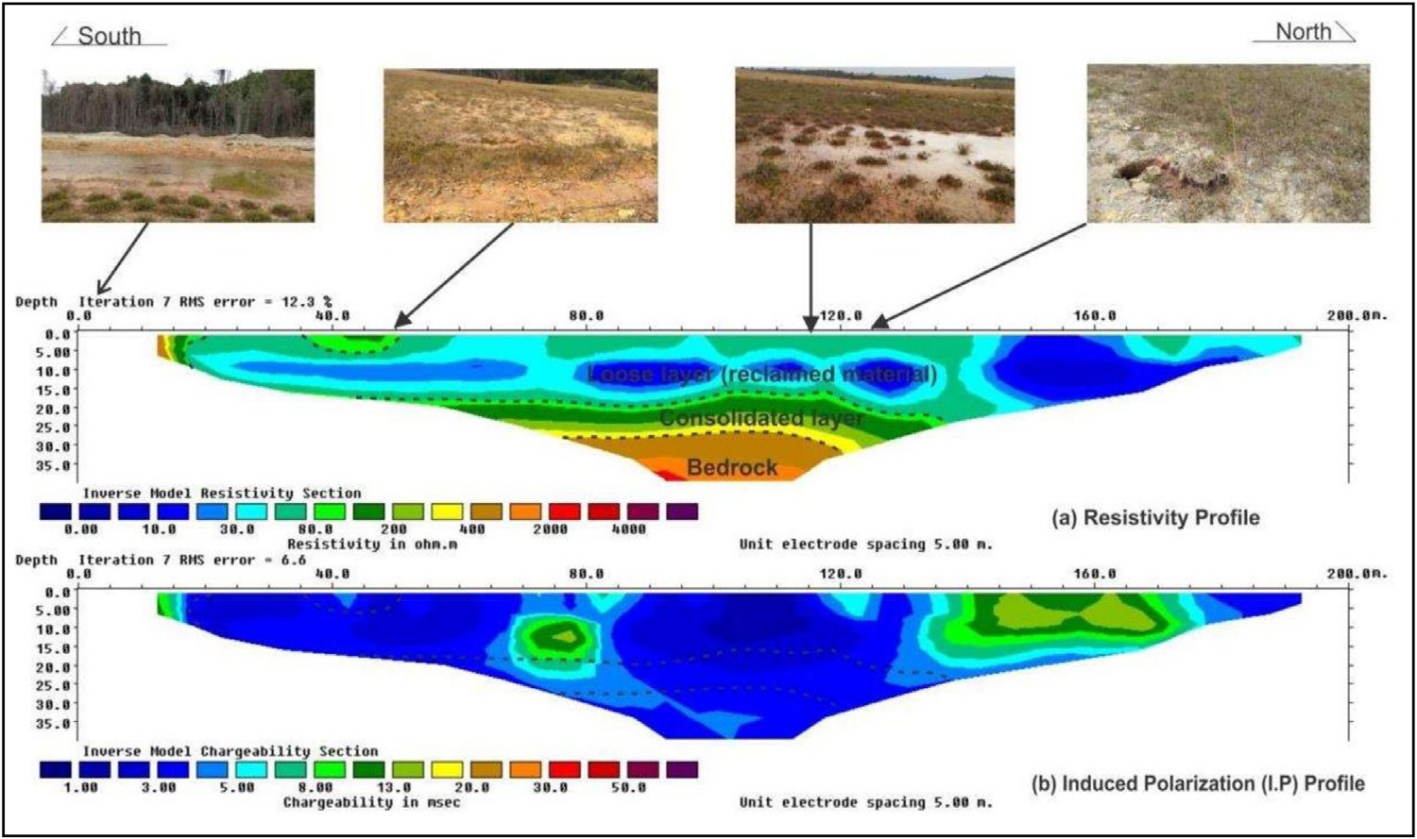
A typical RES2D ERT and IP geophysical profile data showing the three categories of the subsurface lithological layers as represented by the colour codes, and ploted along the survey line.

A typical of the RES2D ERT data obtained along the geophysical survey line as shown in Fig. 4. The three (3) categories of the subsurface lithological layers are represented by the colour codes and plotted along the six profiles. The first layer is designated by dark blue to light blue colours, while the light green to yellow colours represents the second layer. The last layer with the highest RES2D ERT values, was assigned the brown to dark purple colours.

The raw data from measurements along the six (6) RES2D ERT survey lines are presented in the Excel supplementary file. Subsurface classifications of the stratum underlaid the study site was made possible by the RES2D raw data into three major lithological unit layers in sequential order of; (a) the soft layers, that forms the first lithological unit, which covers the IWD and MWM materials. The resistivity values recorded varied from 0 – 100 Ω-m, at varied depths of between about 10 and 15 m. The second lithological unit consist of the consolidated layers which produced subsurface strata with varied resistivity values from about 101 – 400 Ω-m, at varied depths of about 15 - 20 m. The last, and third lithological unit comprises of the bedrock has the highest resistivity values which varied between about 401 and 2000 Ω-m, at depths between 20 - 35 m.

The details survey parameters used in the plotting of 3D Oasis Montaj integrated with the RES2D raw datasets with the GPS readings for each electrode position along the six geophysical profile lines, are presented in the supplementary file lebelled as, the details survey lines parameters. The details survey parameters, together with the inverted RES2D and the GTPS positions of the electrodes, were used to estimate data for the volume of IWD and MWM waste materials as 312,000 m ^3^, using 3D Oasis Montaj modeling through the development of a rectangular prism model generated.

The data Images for the industrial and municipal wastes are presented in the supplementary file that provides clear pictorial images of all the six geophysical survey lines. The images provide details physical assessment of the study site conditions and the effect of industrial and municipal wastes on the ecosystems at the area. The data helps to assess hazardous contaminants plumes from the industrial and municipal environmental wastes as it oozes out from the dumped materials, i.e., the survey line TKI 5 with the worst scenario showing the demarcation of the PEH.

Data from the soil, and water samples collected at the site, were analyzed for hazardous waste materials and presented in the supplementary Excel file format with a view of given as much details of the datasets as possible for the industrial and municipal wastes materials. The parameters from the data showed mixtures of the IWM and MSW at the site as a potential hazardous risk to the environment, human, ecosystems, surface water and subsurface water bearing zones, and the structural features in the area.

## 2 Experimental Design, Materials and Methods

### 2.1 Experimental design

Extensive data acquisition through the subsurface probing by the deployment of surface instruments that helps to map the hazardous materials, and thus, computed contaminants volume emanating from both the IWM, and the MSW, is essential for meticulous data analysis, and management [1]. A RES2D ERT geophysical survey was successfully carried out at the Lot 53360, Majlis Agama Islam Wilayah Persekutuan property, Taman Kepong Indah (TKI), 68100, Kuala Lumpur, Malaysia, with the aim of mapping and quantifying the approximate contaminants volume of the industrial and municipal waste materials dumped in the area. The survey was carried out using ABEM Terrameter SAS4000 together with ABEM LUND ES 10 - 64C, the electronic switching selector unit, Fig. 3, [2,5,6]. Six (6) ERT geophysical survey lines were conducted at the project site, using the Wenner-Schlumberger array to obtain the expected minimum depth of the subsurface stratum at about 35 m. The survey data helped to delineate depth of the industrial, and municipal waste materials, and hence, the contaminants volume of the prism shape generated was approximated [1].

Lithological variant, and large dynamic diversity between the different subsurface stratum, particularly in the data acquisition area, the RES2D ERT geophysical survey was selected, and used, to define the mixtures of the IWM, and MSW, as well as the bedrock, and the subsurface groundwater structural features underlain the Kepong area that could be prone to the contaminants plumes. The six ERT geophysical profiles, consists of a modeled cross-sectional two-dimensional plot of the RES2D data in Ω-m units, against the depth to the subsurface structural lithological layers in metre [4].

Wenner-Schlumberger electrode arrays was selected because of its sensitivity to the subsurface vertical structural variations, and better signal to noise ratio than the other electrical resistivity arrays for shallow features prospection [4]. The ERT field data was modeled using the RES2DINV software [7].

### 2.2 Materials and methods

Data for the six RES2D ERT geophysical survey profiles, were acquired along the E-W directions for the survey lines TKI 1 to TKI 3, while the survey lines TKI 4 to TKI 6 was acquired in the perpendicular N–S directions as shown in Fig. 1. The recorded RES2D data were separated into three (3) categories based on the delineated subsurface earth materials in the Kepong area, (i.e., Fig. 4). The dark blue to light blue colour, indicates lower range of subsurface stratum resistivity values, (i.e., from 0 Ω-m – 100 Ω-m). The light green to yellow colour, represent the intermediate lithological layers with resistivity values in the range of about 100 Ω-m to 400 Ω-m, and the last layer, designated with brown to dark purple colour represents the highest resistivity values that range from about 400 Ω-m to 2000 Ω-m.

To produce a 3-D model that helped in the estimation and quantification of the approximate contaminants volume of the waste materials, the inverted RES2D and IP data files, the GPS data for each electrode position of the six profile lines distributed across the site, were compiled together using the Oasis Montaj Software© [8]. The process is very significant in order to adequately map and quantify the distributions of the hazardous materials, and the leachate contaminants plume flows with the subsurface stratum in the area.

The data acquisition is very crucial as the contaminant plumes instigated hazardous effects on ecosystems, human and environment, and any continuous exposure for a long period could results in the development of associate stern health risks [9].

The E-W, and N-S, directions of the RES2D ERT survey profiles perpendicularly arranged, was decisively designed to facilitate approximate quantification of the waste materials' contaminants volume, whilst the 200 m length of the geoelectrical survey lines, were considered sufficient enough to cover as much segment of the waste materials as possible [1].

The model variables as derived from the subsurface geoelectric parameters, e.g., the thicknesses, resistivity values, and the GPS readings, were integrated using the 3 -D Oasis Montaj software are critical so as to better characterized, and estimated the volume of the contaminants waste mixtures, as shown in Fig. 1.

## Ethics Statement

The authors declare that they have no known ethical issues in respect of the data reported in this article

## Declaration of Competing Interest

The authors declare that they have no known competing financial interests or personal relationships which have, or could be perceived to have, influenced the work reported in this article.

## Appendix Supplementary materials

Image, application 1

Image, application 2

Image, application 3

Image, application 4

Image, application 5
