# Supplementary material for: Data for the industrial and municipal environmental wastes hazard contaminants assessment with integration of RES2D techniques and Oasis Montaj software
Source: Data Brief. 2020 Nov 28;33:106595. doi: 10.1016/j.dib.2020.106595 (PMC7726653; doi:10.1016/j.dib.2020.106595)

Images for the industrial and municipal environmental wastes hazard contaminants assessment


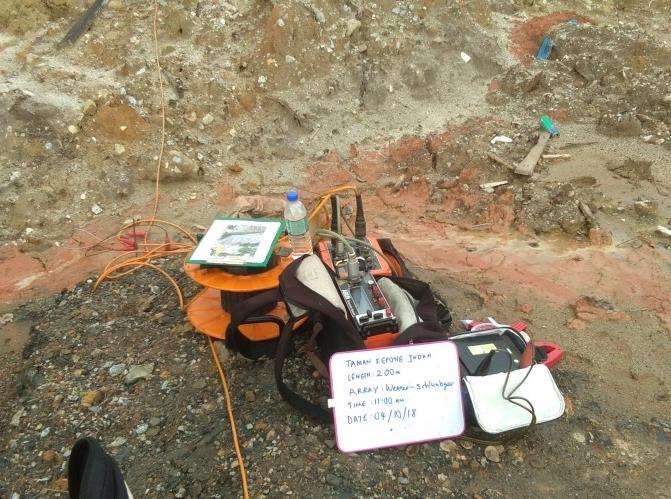

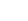

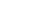

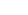

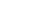

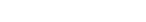

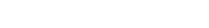

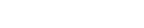

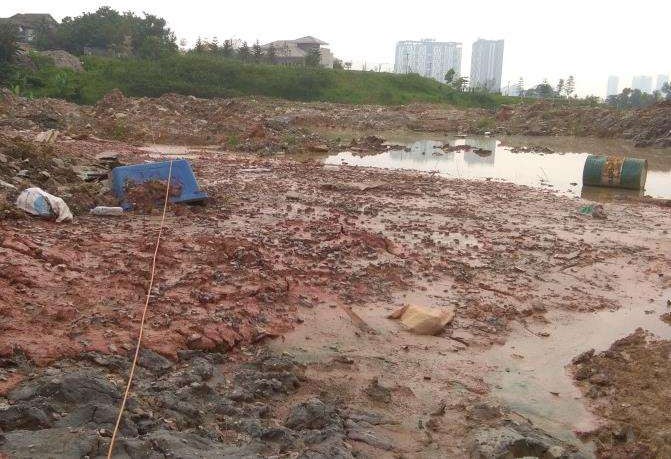

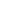

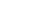

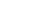

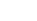

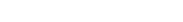

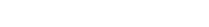

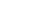

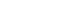

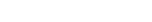

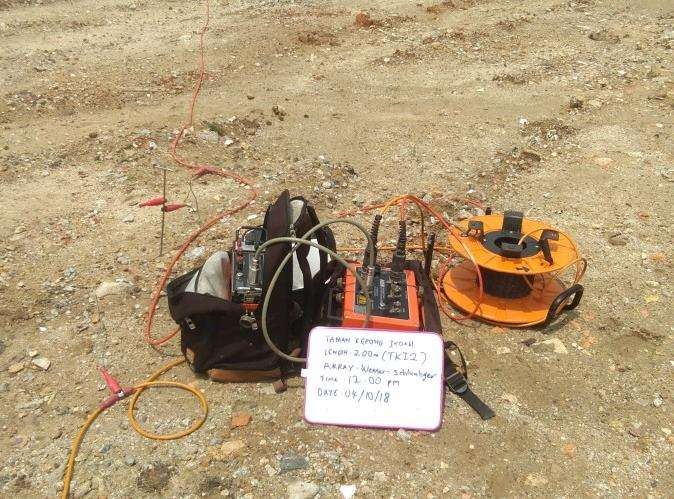

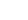

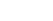

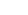

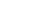

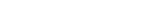

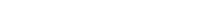

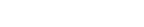

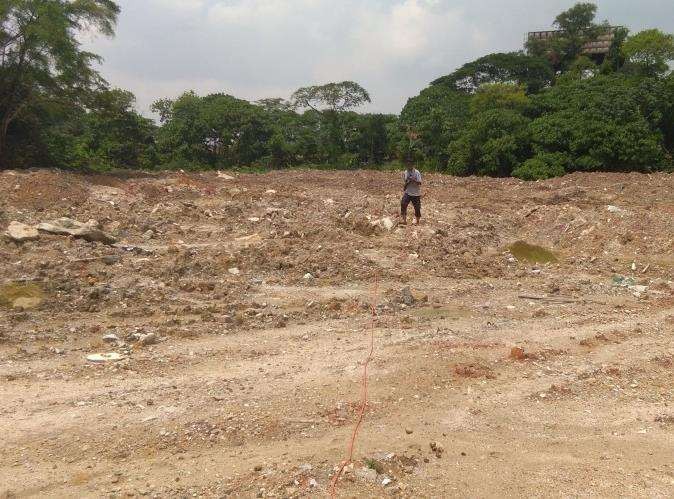

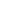

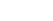

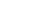

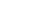

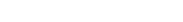

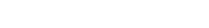

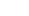

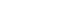

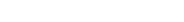

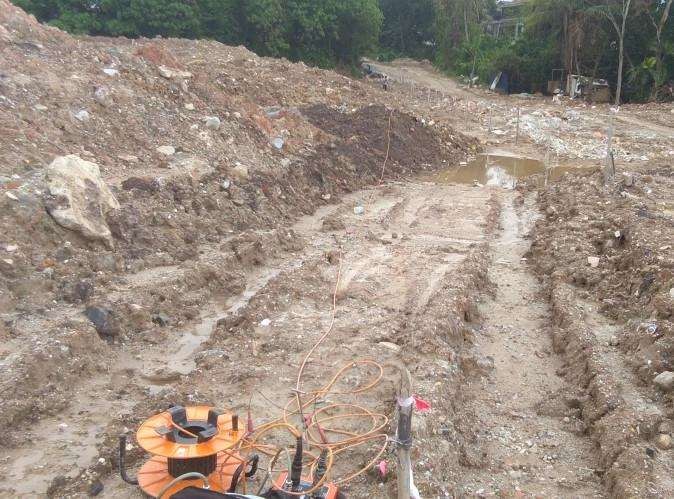

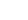

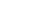

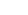

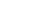

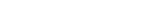

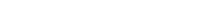

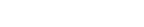

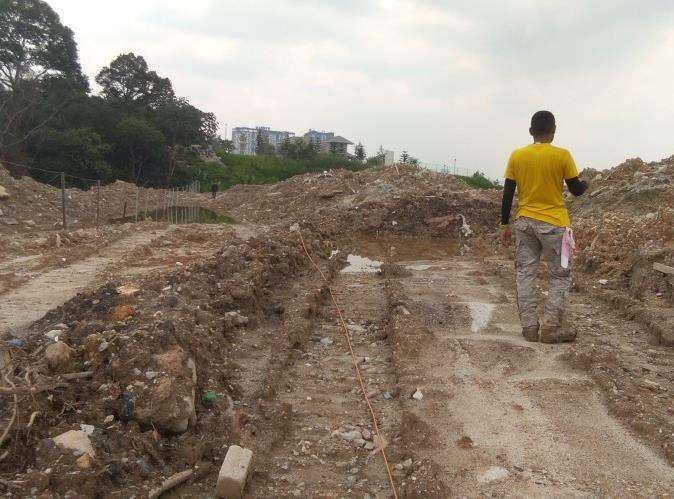

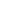

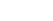

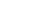

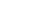

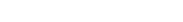

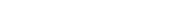

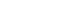

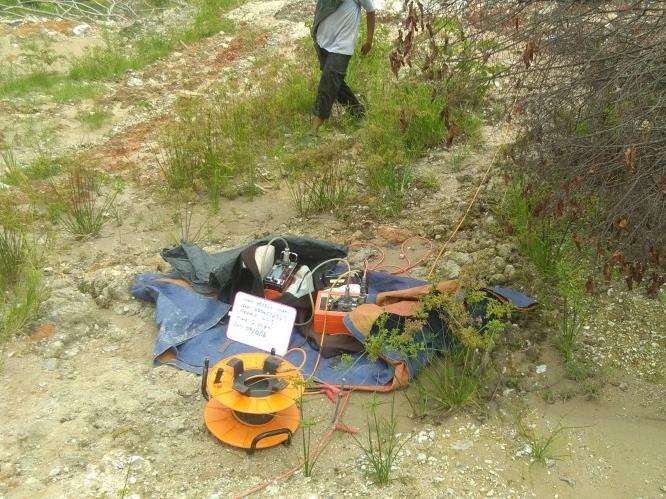

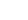

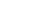

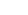

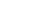

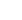

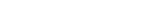

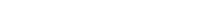

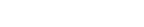

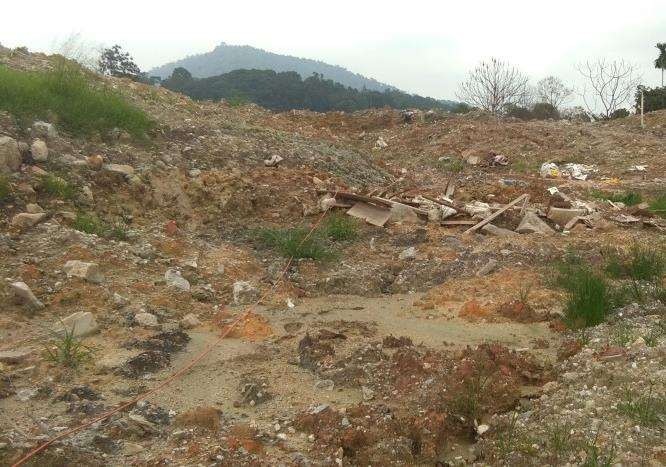

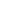

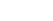

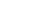

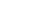

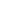

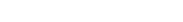

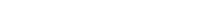

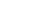

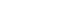

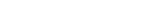

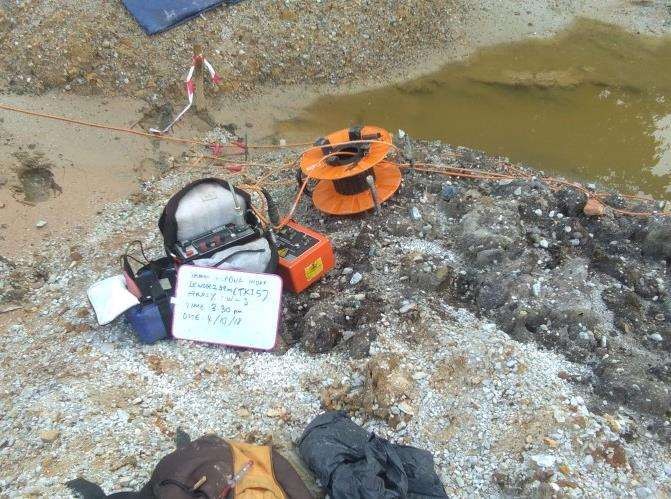

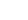

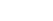

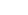

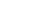

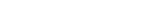

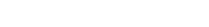

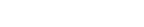

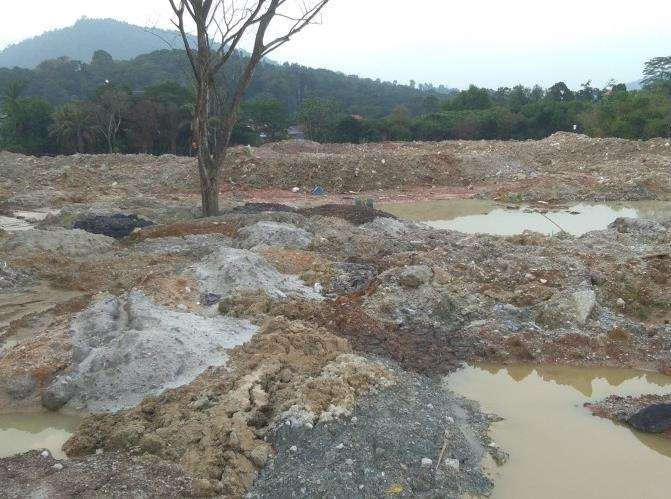

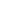

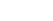

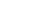

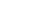

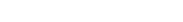

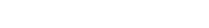

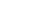

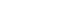

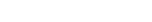

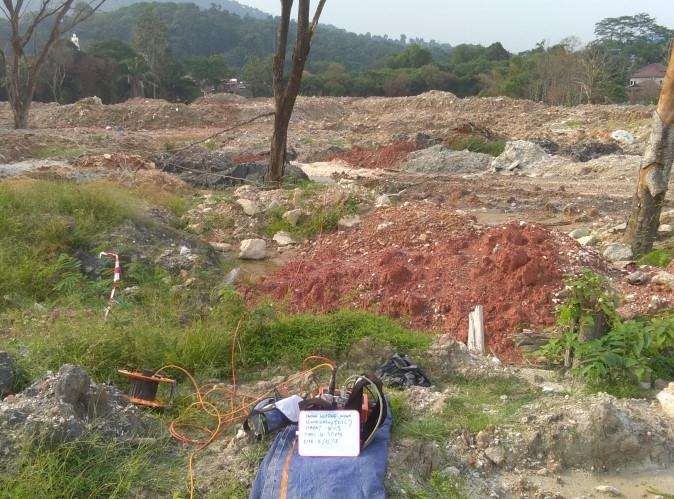

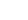

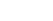

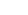

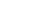

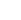

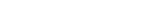

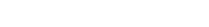

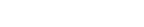

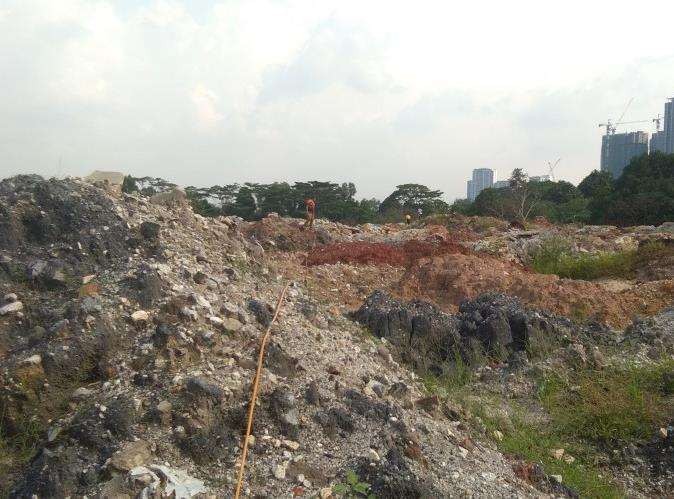

Supplement: Supplementary file 1 [file mmc1.doc]
